# Dental Ethics

**Published:** 1887-10

**Authors:** T. H. Lipscomb


ARTICLE III.

### DENTAL ETHICS.

BY T. H. LIPSCOMB.

In discussing this question, so important not only to us as dentists, but to the world at large as our patients, let us first endeavor to understand clearly the meaning embraced in this term. Ethics is defined to be a code of laws for the government of a body in their conduct toward each other as a body, and toward others with whom they are thrown in contact. Since men in the earliest times began to associate together there has been found the necessity of a code of laws to regulate and control them. These were at first very few and simple, being only what was generally conceded to be the common rights. But later on, as the population of the world increased and men began to form themselves into kingdoms and nations, then a code of written laws came into vogue, and penalties were provided for their violation. Still later on, when men began to seek other means of gaining a subsistence than by tilling the soil, and found a necessity for skilled labor, then they divided themselves into trades and professions, and these were sub-divided into specialties, the need of a code of ethics to regulate the practitioners of each profession in their conduct toward each other and toward their patients was found necessary. This was especially so in the practice of medicine, and dentistry being but a branch of that profession, must feel a like need.

The American Dental Association formulated a code, which is, or should be familiar to each of you, and fills to a great extent the requirements of the profession, and has since been adopted by all of our associations for their governments. It therefore remains with you to enforce this code, as formulated by the American Dental Association and adopted by you, and which you have pledged yourself to upport.

You are all aware of the fact that to-day there exists a greater necessity for the enforcement of these laws than in almost any State in this Union. Why? Because Texas to-day stands almost alone and solitary as one State that has refused to protect her people from the quack and charlatans who being debarred by law from practicing in their native places, come to Texas, pay their occupation tax, and swinging their shingles to the breeze of heaven announce themselves as dental surgeons, and guarantee all their work as first-class. The State of Texas refuses to protect her people from imposters by the enactment of laws forbidding any to practice dentistry who are not graduates of some reputable dental college, or who pass a creditable examination before a board of examiners appointed either by the State or by this Association. This examination should be such a one as any first-class college would demand of its students before conferring on them its diplomas. In the meanwhile, we, members of this Association, should endeavor to protect our people by raising the standard of our Association so high that all will endeavor to enroll themselves under our banner, and let being a member of "The Texas Dental Association", be a recommendation strong enough to satisfy the most skeptical. The higher you raise the standard of our Association the higher it will be appreciated, both by the profession and by the people. A dentist desiring membership in this society should be compelled to undergo such an examination as you may see fit to demand, but it should at least be as rigid as any college demands of her graduates. The aim of our profession should be to raise itself everywhere. Our colleges are working with that end in view, and we should support them in their efforts. Some of them have done away with the five-years clause, and compel their students all to attend two years regardless of previous experience. This will have its effect in time of elevating the standard, and they will have a better theoretical knowledge of their profession, the practical will come in time. Men now in the profession should refuse to take young men as students unless they will pledge themselves to attend college. Perhaps some may criticise my dwelling on higher education, but it is the educated man who takes pride in his profession and strives to raise it by every method in his power, white it is the uneducated dentist who, as a rule, does not feel that pride, or observe the ethics of the profession. It is usually this class of men who resort to printer's ink to draw patients to their office by glaring advertisements. On this subject I will quote from an address by Dr. Geo. J. Friedrichs, before the Louisiana Dental Society:

"Notwithstanding our dental colleges are daily sending forth many worthy and thoroughly competent men, the deplorable fact stares us in the face that a great number of young men enter the profession not properly qualified, and in the great majority of cases the dentists themselves are to blame. To illustrate: a boy is hired to attend the door bell; in his leisure time he assists in the laboratory; eventually he learns to extract teeth; self-conceit steps in, and with swelling pride he concludes that he knows more than his preceptor; or the case may be that he is thrown in contact with some ignoramus practicing dentistry, and he feels his superiority. In either case he hangs out his shingle a full-fledged dental surgeon, though perhaps never having seen the inside of a dental college, or ever read a single treatise pertaining to the practice of dentistry. This is the class which generally produces the advertising dentist; though it must be confessed with shame and sorrow that there are some who have drank at the fount of knowledge, so lost to every honorable impulse, so debased in principles, as to disgrace their alma mater and prostitute their profession for lucre's sake. Advertising dentists! 'By their fruits ye shall know them.' An advertising dentist is not a modest man, for he does not 'hide his light under a bushel;' he is not a contented man, for he publishes to the world that a generous public has not awarded him his due share of employment; he is not an upright man, for he disregards the edicts of his profession in not obeying its code of ethics; he is an unprincipled man, for he tries to raise his own status by belittling the standing of his fellow practitioners; he is an arrogant man, 'for he knows it all.' I pity the man who in his arrogance places himself above being taught. He is a conceited man, for he judges others by his own standard; he is a selfish man, for his whole soul is wrapped up in self, and to him the almighty dollar attracts every spark of honorable merit; and lastly, he is a dishonest man, for he promises results which he knows cannot be attained, thereby obtaining money under false pretenses."

The clause in our code in regard to advertising is very plain and definite, and any infringement on it should be promptly noticed by you. A simple card, announcing name, business and address being all that is allowed. This does not cover putting name, business and address in box-car letters on a twenty foot board, on the fences of our public roads, as some of our professional brothers seem to think.

In regard to the treatment of our dentist toward another, we can only be careful to observe the courtesy which is practiced by gentlemen in polite society. Do not do or say anything in disparagement of a brother dentist; do not criticise his work in presence of his patients. If you cannot say anything in his favor, remember that "silence is golden." Words said intending to reflect on a fellow practitioner will often injure the speaker more than him at whom it was aimed. How often, though, do we see professional envy and bitter feelings crop out, and dentists say things of each other behind their backs which would not be tolerated if said in their presence. Our code says that the young man should show special respect to the old, and the old man should encourage the young; but how often is the reverse of this the case. Our young men, fresh from the walls of their alma mater, will sneer at their elders call them "old fogies," "old moss-backs," etc. Nor are the older men much better. Instead of their taking the young man by the hand, encouraging him and helping him over the hard places, enabling him to get fairly started in his course, they will sneer at their younger rival, dubbing him as a "greenhorn," or as "a brainless school-boy."

In speaking of our conduct in our intercourse with our patients, I cannot do better than quote from an address by Dr. Freeman of the Vanderbilt Dental College, to the class of 1882. He says: "However lofty your attainments may become in your profession, it will still be absolutely essential that you should be a gentleman in the highest sense of the word. Society will have no ornament however sacred, that will not be committed to your keeping. The delicate and refined treasures of this life must sit beneath your shadow and pass under touch of your hands. The physician deals with the sick, but the healthful are entrusted to your confidence. If you bear with you into the riper years of life this sacred trust of confiding society, it will grant you a place among the highest of the high, and crown you with the brightest chaplet to be worn in declining years; but if you, for want of manly principles and care, fail in this important career which you have voluntarily chosen, your name will justily be enshrouded with gloom, and your last retreat will be found among earth's most unworthy. Gentlemen, you must be intelligent, as well as chaste and elegant in deportment, for the best representatives of mental culture are those who will most frequent your office. The uncultivated and uncivil will apply for relief from pain, but their stay will be short and their visits unfrequent; but the affluent and the gifted will seek your chair, and feel disappointed, and go away to return no more if you prove an unworthy peer. The treatment of the most skilled hand is often declined because it is preceeded by an uncultivated tongue, together with forbidding surroundings. Neglect will ofttimes produce misfortune which you are called upon to remedy. These you must see and yet not seem to see. Your patience and gentleness must often bridge for the timid the chasm of imagination or well-grounded fear. Firmness and kindness, encouragement and truthfulness, are connected with each other, and must constantly attend your chair. Courage and sympathy are manly traits, and are the faithful dentist's strongholds. Make your office genial and attractive. Cultivate promptness of comprehension so as to read your patient rapidly, and without any delay advance with your purposes.—*Texas Dental Journal.*

